# Clinician Perspectives on Prescribing Restrictions and Opioid Pain Management in Australian Podiatric Practice: A Qualitative Study

**DOI:** 10.1002/jfa2.70202

**Published:** 2026-08-19

**Authors:** Lachlan Aquilina, Mischa Kronenberg, Estie Kruger, Marc Tennant, Joon Soo Park

**Affiliations:** ^1^ School of Health and Clinical Sciences The University of Western Australia Perth Western Australia Australia; ^2^ Department of Podiatry and High Risk Foot Royal North Shore Hospital Sydney New South Wales Australia

**Keywords:** drug prescriptions, health policy, opioid analgesics, pain management, podiatry, primary health care

## Abstract

**Background:**

Australian‐endorsed general podiatrists and podiatric surgeons (EGPs and EPSs, respectively) operate under a fixed prescribing formulary, the National Podiatry Scheduled Medicines List (NPSML), that restricts access to opioid analgesics including tramadol and tapentadol, despite emerging evidence questioning the efficacy of available alternatives such as codeine. The impact of these restrictions on clinical practice has not been qualitatively examined.

**Methods:**

A qualitative descriptive study using semi‐structured interviews was conducted with thirty‐one clinicians: ten pharmacists, seven dentists, five EGPs and nine EPSs practising across metropolitan, regional and rural Australia. Profession‐specific interview guides explored prescribing practices, analgesic strategies, experiences with formulary limitations and perceived impacts on patients and care systems. Interviews were audio‐recorded, transcribed verbatim and analysed using comparative thematic analysis with inductive coding. This study was reported in accordance with the COREQ checklist.

**Results:**

Three themes were identified: (1) Inadequacy of fixed prescribing formularies: All professional groups, including pharmacists and dentists not directly constrained by the NPSML, critiqued the static, list‐based prescribing model as structurally misaligned with contemporary evidence and practice. (2) Codeine as an unreliable ceiling for moderate to severe pain: Codeine was widely regarded as an unreliable analgesic because of variable metabolism, with clinicians describing cases of treatment failure necessitating emergency department referral. (3) Fragmented care pathways and cascading patient impact: Prescribing restrictions were perceived to generate disjointed care pathways, delayed pain relief, increased healthcare utilisation and disproportionately affected patients in rural settings.

**Conclusion:**

Australia's NPSML is perceived by clinicians to be structurally misaligned with current pharmacological evidence, professional competencies and equitable care principles. These findings support the transition to a dynamic, scope‐of‐practice‐based prescribing model that can respond to emerging evidence and meet the analgesic needs of diverse patient populations.

## 1 Introduction

Pain management is a critical aspect of podiatric care, with analgesics cited as one of the most commonly prescribed medication groups by Australian‐endorsed general podiatrists and podiatric surgeons (EGPs and EPSs, respectively) [1]. Currently, EGPs have authority to prescribe codeine as a combination product with paracetamol, and EPSs additionally have authority to prescribe oxycodone as an immediate‐release product. The regulatory landscape in Australia restricts prescribing authority for opioids with high clinical utility, such as tramadol, morphine and tapentadol, among podiatric practitioners. There remains a scarcity of research investigating the impact of these restrictions on clinical practice.

Codeine, although the only opioid available to EGPs, has well‐documented limitations; its efficacy is uncertain, with evidence suggesting that paracetamol alone may be equally effective [2, 3]. Tramadol has historically been considered more efficacious for moderate to severe pain and has been associated with a lower risk of respiratory depression, reduced dependence and greater dosing flexibility [4, 5, 6, 7]. However, its safety profile has undergone significant re‐evaluation, with concerns regarding increased risk of all‐cause mortality, cardiovascular events and fractures in chronic pain populations [8]. Tramadol also carries a unique risk of serotonin syndrome when co‐prescribed with CYP2D6‐inhibiting antidepressants [5]. Tapentadol, when compared to oxycodone, has been considered to offer lower rates of constipation and potentially lower abuse potential, though it too carries serotonin syndrome risk and remains outside the National Podiatry Scheduled Medicines List (NPSML) [9, 10, 11, 12].

Despite the evolving evidence base, the prescribing competencies required to safely prescribe tramadol or tapentadol are unlikely to differ substantially from those required for codeine and oxycodone. The Podiatry Board of Australia, via AHPRA, provides endorsement for scheduled medicines upon completion of approved training; however, prescribing authority itself is conferred and regulated through state and territory poisons legislation, which determines whether endorsed practitioners may prescribe, possess, supply or administer specific scheduled medicines. Irrespective of this jurisdictional legislative access, endorsed practitioners remain bound by the NPSML as the applicable prescribing formulary. Australian dentists, who occupy an analogous role, have authority to prescribe a broad range of opioids under a scope‐based model, and pharmacists provide a further perspective on prescribing practices across multiple professional groups. This study sought to answer: How do NPSML restrictions on opioid prescribing affect clinical decision‐making and continuity of care across EGPs, EPSs, dental clinicians and pharmacists in Australia?

## 2 Methods

### 2.1 Study Design

This study used a qualitative descriptive design, drawing on semi‐structured interviews to explore clinicians' perspectives on pain management and prescribing restrictions. This study focused on how NPSML limitations, particularly regarding tramadol and tapentadol, influence clinical decision‐making, continuity of care and perceived patient outcomes across four professional groups: EGPs, EPSs, dentists and pharmacists. This study is reported in accordance with the Consolidated Criteria for Reporting of Qualitative Research (COREQ) checklist.

### 2.2 Ethics Approval

Approval was obtained from the University of Western Australia Human Research Ethics Committee (HREC 2024/ET001152).

### 2.3 Researcher Positionality and Reflexivity

The primary researcher (LA) is a registered EGP. This insider perspective provided familiarity with the clinical and regulatory landscape but carried a risk of interpretive bias; notably, LA holds the prior view that the NPSML is overly restrictive and impedes optimal patient care. To manage this, JSP, a dentist, pharmacist and credentialled diabetes educator, oversaw all coding decisions, providing an external check against confirmation bias throughout the analytical process. Six of the thirty‐one participants were known to LA prior to the study; no perceived power dynamics were at play, and to manage this, participants were explicitly advised at the outset of each interview that their responses would have no bearing on any professional or personal relationships with LA and that participation was entirely voluntary and confidential. Reflective journalling was maintained by LA throughout data collection and analysis. Peer debriefing was conducted via biweekly telephone calls between LA and MK, a senior non‐endorsed general podiatrist, who provided an independent perspective and challenged emerging interpretations.

### 2.4 Participants and Setting

Participants were registered health professionals practising in Australia. Eligibility criteria required participants to be either an EGP, an EPS, a dentist or dental specialist, or a pharmacist, all of whom held current AHPRA registration. Podiatrists and podiatric surgeons without endorsement for scheduled medicines were excluded. Recruitment was conducted through social media platforms (Facebook and LinkedIn), professional networks and professional organisations, including the Australasian College of Podiatric Surgeons and the Australian Podiatry Association. Snowball sampling was additionally employed to extend reach beyond initial contacts. Because of the nature of recruitment channels utilised, the total number of clinicians invited or who declined participation could not be determined.

### 2.5 Data Collection

Semi‐structured interviews were conducted using profession‐specific interview guides exploring common domains: types of pain encountered, analgesic strategies, experiences with prescribing limitations, alternative strategies when preferred medicines were inaccessible and perceptions of patient, clinician and system‐level impacts. Interviews were undertaken via MS Teams, recorded, transcribed verbatim and de‐identified. There was no third‐party presence for any interview, nor were any repeat interviews conducted. Participants were assigned professional identifiers (e.g., EGP 1 and Dentist 3) to maintain confidentiality. All interviews were conducted by LA (male) between 14 April 2025 and 4 November 2025, with interview duration ranging from 33 minutes to 1 hour and 12 minutes. JSP reviewed all recorded interviews prior to transcription.

### 2.6 Data Saturation

Data saturation was assessed retrospectively by monitoring the emergence of new themes across successive interviews. Saturation was considered achieved when no new themes or subthemes were identified across three consecutive interviews. All three themes were represented across all four professional groups. These findings are consistent with empirical research indicating that 80%–92% of themes are typically captured within the first ten to twelve interviews [13]; the total sample of thirty‐one clinicians substantially exceeds this threshold.

### 2.7 Data Analysis

Comparative thematic analysis was undertaken. Transcripts were read iteratively to identify recurring patterns within and across professional groups. LA and JSP conducted manual, inductive line‐by‐line coding, generating descriptive codes grouped into themes based on patterns of convergence. Attention was paid to areas of convergence and divergence among groups, especially where differences reflected professional scope, regulatory frameworks or access to medicines. Verbatim quotations are presented with professional identifiers. See Table 1 for the coding tree in summary form.

**TABLE 1:** Coding Tree in Summary Form: Three themes, fifteen categories and forty‐seven first‐order codes.

Theme	Category	First‐order codes (condensed)
Theme 1: Inadequacy of fixed prescribing formularies	1.1 Static list architecturally misaligned with evidence	Fixed list frozen at enactment; requires legislative amendment to update; cannot respond to emerging evidence; list not reviewed for years; out of date
	1.2 Codeine/oxycodone permitted; tramadol/tapentadol prohibited—logical inconsistency	S4 tramadol restricted, S8 oxycodone permitted; same drug class but differential access; allows risky drug, denies safer option; arbitrary drug selection; no pharmacological rationale
	1.3 Guideline/formulary misalignment compels off‐guideline prescribing	eTG recommends tapentadol; NPSML permits oxycodone only; prescribing second‐line by regulatory compulsion; forced to deviate from best‐practice guidance
	1.4 Scope‐of‐practice‐based model preferred across all groups	Open formulary preferred; nurse practitioner model cited favourably; prescribing should track competence, not a list; optometry model referenced; scope should determine formulary
	1.5 Therapeutic guidelines should dynamically inform prescribing authority	eTG should drive formulary updates; evidence‐based prescribing requires dynamic governance; annual review recommended; policy should track guidelines
Theme 2: Codeine as an unreliable ceiling for moderate to severe pain	2.1 Pharmacogenomic variability renders codeine clinically unpredictable	Prodrug metabolism; poor/ultrarapid metabolisers; ∼30% no efficacy; variable effect across ethnicities; prodrug conversion failure; codeine ‘a coin flip'
	2.2 Codeine absent from or deprecated in current therapeutic guidelines	Not recommended by eTG; removed from guidelines; replaced by paracetamol/ibuprofen in dental guidelines; not first‐line; eTG recommends tramadol over codeine
	2.3 Clinical failure of codeine documented by all professional groups	Codeine prescribed; patient still in pain; escalation required; codeine ineffective post‐procedure; patient in tears; sent to ED; anecdotal failure across all groups
	2.4 Oxycodone as a suboptimal substitute when codeine fails	Oxycodone only option beyond codeine; oxycodone overly strong for moderate pain; constipation/addiction risk; cognitive impairment; ‘too heavy’; pharmacist interrogation delays
	2.5 Tramadol and tapentadol identified as superior alternatives for moderate pain	Tramadol preferred over codeine; tapentadol preferred over oxycodone; multimodal mechanism; lower abuse potential; fewer adverse effects; eTG preference for tapentadol; clinical familiarity in hospital settings
	2.6 Tapentadol preferred over oxycodone by podiatric surgeons for post‐surgical pain	Tapentadol used by anaesthetist instead; immediate and slow‐release formulations needed; better tolerability; lower opioid dependency risk; preferred by surgeons; tapentadol already prescribed for surgical patients
Theme 3: Fragmented care pathways and cascading patient impact	3.1 Necessity‐driven referral for opioid analgesic as a routine clinical workaround	Refers to GP for tramadol; contacts anaesthetist for tapentadol; shared care arrangement required; emergency call to anaesthetist at night; systematic workaround described
	3.2 Delayed pain relief and consequent patient suffering	Waiting hours in ED for pain relief; unmanaged post‐op pain overnight; delay in obtaining script; patient chasing pain; suffering unnecessarily; inefficient use of patient time
	3.3 Loss of care continuity and erosion of patient trust	GP disparages podiatry profession; patient lost to follow‐up; patient confused about roles; patient blames podiatrist; loss of professional credibility; ‘can't prescribe your own patient'
	3.4 Disproportionate rural and remote impact	GP appointments 2 months away; ED wait 6 hours; rural patients most disadvantaged; no bulk billing for GP visit; distance to access; prescribing restriction amplified by geography
	3.5 System‐level healthcare burden: ED, Medicare and after‐hours	Unnecessary ED presentations; overloading ED; Medicare inefficiency; after‐hours GP visit; ED doctors frustrated; costs borne by system; public resources misused
	3.6 Undermined professional credibility and autonomy	Patient asks ‘why can't you prescribe this?’; surgeon cannot prescribe own patient's post‐op analgesia; looks incompetent; nurse practitioners have greater access; AHPRA complaints linked to formulary
	3.7 Training and competency perceived as already adequate for expanded prescribing	Endorsed prescribers already manage opioids; no additional training required; CPD sufficient; online module adequate; endorsement competencies transfer; pharmacy check as safety net

## 3 Results

A total of thirty‐one clinicians were interviewed, comprising ten pharmacists, seven dentists, five EGPs and nine EPSs, representing a range of clinical experience and practice settings across private, public, academic and mixed models of care—see Table 2. Clinicians described broadly similar principles guiding pain management but markedly different experiences of constraint, autonomy and responsibility arising from prescribing frameworks. Although all groups emphasised conservative, stepwise analgesic approaches, the extent to which clinicians could independently manage moderate to severe pain varied substantially. Three predominant themes were identified.

**TABLE 2:** Participant work environments.

Participants	Reported setting/role
EGP 1	Sports medicine clinic
EGPs 1, 2 and 5	General podiatry clinic
EGP 3	Rural general podiatry clinic
EGP 4	Public hospital
EPSs 1–9	Private surgical practice
EPS 9	Rural private surgical practice
Dentists 1, 2, 4, 6 and 7	Unspecified
Dentist 3	Public hospital
Dentist 5	Private dental clinic
Pharmacists 1–3, 5–7, 9, 10	Unspecified
Pharmacist 4	Private pharmacy
Pharmacist 8	Public hospital

### 3.1 Theme 1: Inadequacy of Fixed Prescribing Formularies

The most universally pervasive theme across all professional groups centred on the fundamental inadequacy of static medication lists that fail to evolve with evidence and practice needs. Rather than a complaint about specific drugs being missing, this was a critique of the list‐based model itself as structurally misaligned with contemporary healthcare.

Pharmacists expressed the most systematic criticism, describing the NPSML as emblematic of poor regulatory design:

It's a terrible, terrible approach, locking in a fixed list that's frozen in time and needs to be updated in legislation to be able to update it, because you can't adjust and change to prescribe in line with quality use of medicines, in line with current best practice

(Pharmacist 1)

Another highlighted what they thought was paradoxical—the nature of the restrictions on EPSs:

It doesn't sound like a very well thought out prescribing framework… you can't prescribe safer and more effective medications, but ones that have evidenced poorer outcomes or aren't recommended are ok to prescribe? tramadol is an S4 and oxycodone is an S8, so you're restricted to prescribe an S4 from the same class of drugs, but it's an S4 for a reason, that's because it poses less risk

(Pharmacist 6)

A third pharmacist identified a perceived fundamental governance problem, noting that a prescribing framework should not be static:

I do think that if there is emerging evidence that suggests that alternatives could offer better efficacy… then the prescribing list or authority should be reviewed… A framework is not set in stone. It should be constantly reviewed pending emerging evidence

(Pharmacist 2)

This sentiment was shared across all disciplines:

If we're capable to prescribe oxycodone, then we should be able to prescribe pretty much anything (other opioids)

(EPS 2)

I think you would have to be pretty competent at prescribing opioids to be able to safely prescribe either of these medicines (oxycodone and tapentadol)… they're more similar than they are different

(Dentist 7)

EPSs articulated their perception of incompatibility between clinical scope and prescribing authority:

If we're going to have a license… to perform, essentially, orthopaedic procedures on the foo… we need to have the right toolbox to look after the patient… they're letting us have full scope surgery… but then they're not following up with the other necessary means with the medicine

(EPS 1)

EGPs described the NPSML as actively suppressive of professional development and scope:

I don't understand why… the list hasn't been updated. I don't even know why we use a list, especially such a narrow one. Why can't we have an open‐formulary, like scope of practice‐based model like nurse practitioners? Being restricted really makes me feel like my options are limited

(EGP 3)

So, we've got a formulary that I think is antiquated. It's very much focused towards what a private practitioner might require, rather than an advanced scope of practice practitioner, but it also pigeon holes that private practitioner to work a really basic scope that isn't attuned to modern podiatric practice

(EGP 4)

All professional groups converged on the view that fixed formularies are fundamentally inadequate. The problem that was perceived extended beyond missing specific drugs to the inability of the system itself to incorporate new evidence without legislative amendment, which clinicians viewed as a structural barrier to best‐practice prescribing.

### 3.2 Theme 2: Codeine as an Unreliable Ceiling for Moderate to Severe Pain

Codeine was frequently described as an unreliable or insufficient option for managing moderate pain. This perception was particularly prominent among EGPs, for whom codeine represented the upper limit of in‐scope opioid prescribing.

One EGP observed what they thought to be a fundamental clinical limitation:

Our options are severely limited in terms of opioids and sort of equivalent options… codeine has its limitations… and it won't work for everyone, and so we are somewhat hamstrung when it comes to that scenario

(EGP 2)

Another EGP recalled a direct failure to provide adequate post‐procedural analgesia:

I gave her a script for paracetamol and codeine after the procedure… and it did absolutely nothing for her. Once her anaesthetic wore off, she was in so much pain, in tears, and it was such an awful feeling… I just had to refer her to ED. And it just made me feel so awful, she's in tear inducing pain and waiting for hours and hours in an ED waiting room, waiting for a script for a drug that I could have safely prescribed for her

(EGP 3)

A pharmacist and EGP both captured the absence of guideline support for codeine and their recognition of superior analgesic alternatives:

I probably wouldn't ever choose codeine as a first choice if I was a prescriber. Generally, tramadol would probably be more of an option for moderate or strong pain as like a stepwise approach… therapeutic guidelines recommend tramadol over codeine‐based formulations as well

(Pharmacist 3)

I do it (manage acute, moderate to severe pain) probably inappropriately or inefficiently at the moment… If someone presents with moderate to severe post‐op pain, I'll whack them onto paracetamol and codeine… that would represent me doing it inappropriately perhaps, because that doesn't really fall anywhere into guideline‐based management of pain in Australia

(EGP 3)

Dentists attributed codeine variability to pharmacogenomic differences, noting that its prodrug nature renders it ineffective in a substantial proportion of patients:

Codeine is pretty much a drug I never use because… it's like 30% of people don't actually have the enzyme to convert it, because it's a pro‐drug… it doesn't actually work very well for patients

(Dentist 1)

One EPS described the practical consequences when inadequate analgesia forces escalation of care after hours:

Yeah, we have to co‐manage with GPs and anaesthetists… Sometimes the anaesthetist won't be available and it's after hours, so the GP isn't available either. What do we do there? Send them to after hours (Emergency department) and load up the waiting time for actual emergencies?

(EPS 5)

### 3.3 Theme 3: Fragmented Care Pathways and Cascading Patient Impact

Prescribing restrictions were thought to create disjointed care pathways that compromised pain management, patient satisfaction and healthcare system efficiency. It was perceived that the inability to prescribe preferred analgesics forces patients into multiple transitions of care, each carrying clinical, logistical and financial risks.

EGPs described the systematic impact of necessity‐driven referral:

What the restriction does, is it makes the care a bit less streamlined and it's a bit more disjointed… you should be able to relatively simply provide pain relief when its needed. Instead, it becomes a bit more complicated… they're having to have more medical appointments than need be, and having to gain more opinions from more practitioners, which slows down the process… It adds additional costs to their care… It makes the care process more complicated than need be

(EGP 1)

Another EGP articulated their thoughts about system‐level risk when complex patients transition across multiple providers:

We've got a patient who is transitioning from an outpatient setting, into the ED, onto a ward and back home again… with multiple transitions of care that patient is experiencing different teams each time… the risk for the patient is that things are missed… there is real risk of fragmentation of care when you have to transition and escalate

(EGP 4)

Multiple clinicians independently described a recurring access failure scenario that had become a recognised clinical reality:

I've always argued that it's the Friday afternoon, four o'clock patient where the need is almost the most obvious, because they're not going to be able to get into a GP. Their option is an emergency department, which may or may not require six hours wait… if they have to go all weekend and suffer acute pain… that's obviously going to have its own consequences for the patient's well‐being

(EGP 2)

The referral‐based pathway was thought to create cascading system costs, extending beyond individual patient inconvenience to Medicare inefficiency:

If I have done a nail or wart surgery and they are texting me saying they're in severe pain and I've exhausted codeine as a rescue, I'll get them in for a long‐acting block to try to at least get them a good night's rest… That's pretty inefficient for both me and the patient. Then alternatively… I would refer them to an urgent care clinic or ED, which is inefficient for the patient, its inefficient for Medicare and its inefficient for the medical service that is already overloaded

(EGP 3)

The impact was perceived as disproportionately severe in rural settings, where the structural inadequacy of the NPSML compounded existing access barriers:

Being in a rural setting makes it really hard too, because GP appointments are two months out for patients and ED wait times are six hours long. So where does that leave someone?

(EGP 3)

Especially in a rural or remote setting where it's tough to see practitioners… it's just causing more work for everyone… So, if you aren't able to prescribe it, that just means another trip to a GP in a rural setting, which also is not very possible, so it'd be potentially waiting in urgent care for hours or just not bothering and just dealing with the pain instead, which is suboptimal for a lot of our population

(Pharmacist 5)

EPSs additionally reported that NPSML restrictions undermined patient confidence and professional credibility:

If the patient's in a great deal of discomfort and you're unable to manage them, they lose faith in the surgeon (podiatric surgeon), and they have a negative experience, and it doesn't matter if you have a good outcome of the procedure, it's generally a negative experience for the patient. They remember the pain, not the surgery

(EPS 1)

### 3.4 Divergent Positions & Minority Views

Although support for broader podiatric prescribing rights was the dominant view, several minority positions added important nuance to the analysis.

One pharmacist considered the absence of tramadol and tapentadol less of a detriment in the context of short‐term post‐surgical prescribing and lack of confidence in scope‐of‐practice‐based prescribing model among podiatric practitioners.

It’s not a massive detriment… I’m unsure… if the evidence is enough to justify this flexibility (scope of practice prescribing)

(Pharmacist 8)

For one EPS, the issue was less pressing because analgesia was often managed through an anaesthetist and the procedures they undertook did not frequently result in higher pain levels.

The types of procedures that I’m doing… are fairly low… low pain type procedures. So, I’m not having patients waking up with extreme pain and prolonged pain. Its usually manageable with other means… I can’t see it changing my practice all that much

(EPS 2)

One dentist was conservative in their view of stronger opioids and inclined to defer prescribing responsibility.

I seldom prescribe either, I’d rather liase with the regular medical practitioner or ask the anaesthetist to prescribe thus delegating the responsibility

(Dentist 4)

Finally, an EGP stressed a safety‐focused caveat around monitoring, complications and changes to rescue systems.

There is also a significant amount of work that would need to be done. In managing any complications that arise, particularly around agitation that might happen around the initiation of the drug… How to use those safety systems and by that I mean real time prescription monitoring. And how to monitor patients to ensure that there is no risk of abuse of the drug as well… And whilst naloxone isn’t great for tramadol and tapentadol, its better than nothing and curiously, our current formulary only allows for injected naloxone with podiatric surgeons

(EGP 4)

## 4 Discussion

### 4.1 Statement of Principal Findings

This qualitative study identified three interconnected themes illustrating how fixed prescribing formularies are perceived to create structural barriers to best‐practice pain management in Australian podiatric care. The perceptions identified highlight that clinicians feel the regulatory architecture is misaligned with contemporary pharmacological evidence, professional competencies and the principles of patient‐centred care. Accountability without corresponding authority creates structural inefficiency when clinicians are responsible for managing pain but are not authorised to prescribe the medicines required to treat it. Collectively, these themes directly address the study's research question by demonstrating that NPSML restrictions on opioid prescribing were perceived to constrain clinical decision‐making and fracture continuity of care for EGPs and EPSs.

### 4.2 Regulatory Misalignment and the Case for Formulary Reform

The unanimity with which all four professional groups, including pharmacists and dentists not directly constrained by the NPSML, perceived the list‐based prescribing model as fundamentally inadequate is significant. It represents a perspective that transcends professional self‐interest; pharmacists and dentists independently perceived the same structural problems as EGPs and EPSs. The critique centred not only on the absence of specific agents from the NPSML but also on the inability of the system itself to incorporate emerging evidence without legislative amendment. This finding is consistent with the Australian Government's 2024 Scope of Practice Review, which identified regulatory inconsistencies in prescribing rights across allied health professions and recommended reforms to resolve them [14]. Similar themes of regulatory constraint suppressing clinical autonomy have been identified in qualitative studies of nurse prescribers internationally, where fixed formularies were found to undervalue prescriber expertise and impede patient care [15].

The paradox of permitting EPSs to prescribe oxycodone (Schedule 8) while restricting access to tramadol (Schedule 4) was perceived as a logical inconsistency in regulatory design. The prescribing competencies required for tramadol and tapentadol are unlikely to differ materially from those required for oxycodone, and the mechanisms of action are well within the scope of training for endorsed practitioners. That dentists operate under a scope‐based model with access to a wider and more dynamic range of these agents, whereas podiatrists are confined to a static list, underscores the regulatory disparity rather than any defensible difference in clinical competency. One pharmacist captured this most directly, voicing that practitioners were being compelled to prescribe agents no longer recommended by guidelines while being denied access to those that guidelines support, a position that promotes poor prescribing decisions rather than safe ones.

### 4.3 The Evolving Role of Tramadol

The progressive exclusion of tramadol from Australian Therapeutic Guidelines during the period from this study's inception to completion exemplifies the perceived core problem with static formularies: They cannot accommodate evolving evidence in either direction. The regulatory inconsistency therefore persists: If oxycodone is deemed appropriate for EPSs for acute post‐surgical pain, the case for excluding tramadol and tapentadol becomes difficult to sustain on pharmacological grounds alone.

### 4.4 Clinician Views on Codeine

Participants' characterisation of codeine as an unreliable analgesic ceiling is strongly supported by the pharmacological literature [16]. The clinical scenarios described by participants, patients in significant post‐procedural pain for whom codeine proved ineffective and who subsequently required emergency department presentation, represent a tangible patient safety concern. These accounts align with broader qualitative research on prescribing restrictions, in which clinicians describe being compelled to provide suboptimal care because of regulatory constraints rather than any limitation in their competence or knowledge [15].

### 4.5 Fragmented Care and System‐Level Consequences

The third theme, perception of fragmented care pathways and cascading patient impact, extends the implications of formulary restrictions beyond individual patient encounters to the healthcare system. Participants described a consistent pattern: When in‐list analgesic options proved insufficient, patients were redirected through GP consultations, emergency departments or after‐hours services, each transition introducing delays, additional costs, clinical risks and loss of continuity. This finding resonates with research showing that restrictions on non‐medical prescribers generate downstream healthcare utilisation that offsets any intended benefit of the restriction [1].

The perceived impact was described as disproportionately severe in rural and remote settings, where GP access is already constrained and emergency department wait times are prolonged. The Australian Government's Scope of Practice Review has recognised this inequity and recommended that prescribing reform be prioritised for rural and remote practitioners [14]. The findings of this study add multiprofessional qualitative evidence to that recommendation, grounding it in the lived clinical experience of four professional groups.

### 4.6 Strengths and Limitations

This study draws on a diverse sample of thirty‐one clinicians across four professional groups and multiple practice settings, providing convergent perspectives that strengthen the credibility of the findings. The inclusion of dentists and pharmacists as comparator groups enabled triangulation of perspectives beyond those directly affected by formulary restrictions, and the convergence of views across groups that operate under different regulatory frameworks is a particularly strong validation of the underlying clinical logic. However, participants were self‐selected and may hold stronger views on prescribing restrictions than the broader professional population. The sample excluded regulatory stakeholders and consumer perspectives, which would provide additional dimensions to the findings. The qualitative design captures perceived rather than measured outcomes, and clinical scenarios represent participant accounts rather than verified adverse events; thus, any declaration of impact on patients is anecdotal. Detailed demographic data were not systematically collected, limiting contextualisation of individual responses. Member checking was not conducted, though use of verbatim quotations and convergence across four independent professional groups provide a degree of internal validation. An additional area that would increase transparency is greater clarity around the regulatory decision‐making process in determining the NPSML.

### 4.7 Implications for Policy, Practice and Research

These findings support a transition from a static list‐based prescribing model to a scope‐of‐practice framework that aligns prescribing authority with demonstrated competency and clinical need. The evolving evidence base for tramadol [17, 18, 19], including emerging signals of harm in chronic pain populations, does not resolve the regulatory paradox; rather, it underscores the need for a dynamic model capable of responding to new evidence as it emerges. Future research should incorporate regulatory stakeholder and patient perspectives and evaluate patient‐level outcomes associated with formulary restrictions using administrative health data. Research examining the downstream ED presentations and healthcare costs attributable to NPSML‐driven referrals would provide the quantitative evidence base needed to complement these qualitative findings. Additionally, research on the impact of lack of Pharmaceutical Benefits Scheme access for podiatric practitioners would prove useful.

### 4.8 Conclusions

Australia's NPSML is perceived by clinicians to be structurally misaligned with contemporary pharmacological evidence, clinical competency and the principles of equitable, patient‐centred care. These perceptions further suggest that the inability of the formulary to evolve without legislative amendment compels practitioners to prescribe agents of uncertain efficacy while being denied access to clinically appropriate alternatives. It is further perceived that the resulting care fragmentation disproportionately affects patients in rural and remote settings and generates downstream healthcare utilisation that undermines system efficiency. Importantly, the evolving evidence base for tramadol does not resolve this paradox—it reinforces the case for a dynamic, scope‐based prescribing model that can respond to new evidence as it emerges, rather than one frozen at the point of legislative enactment. These findings support the recommendations of the 2024 Scope of Practice Review and add multiprofessional clinician voices to the case for transitioning from a static list‐based model to a scope‐of‐practice framework aligned with demonstrated competency and clinical need.

## Author Contributions

**Lachlan Aquilina:** conceptualization, data curation, investigation, analysis, writing – original draft, writing – review and editing. **Mischa Kronenberg:** writing – review and editing. **Estie Kruger:** writing – original draft, writing – review and editing. **Marc Tennant:** writing – original draft, writing – review and editing. **Joon Soo Park:** formal analysis, methodology, writing – review and editing.

## Funding

The authors have nothing to report.

## Ethics Statement

Ethical approval was obtained from the University of Western Australia Human Research Ethics Committee (HREC 2024/ET001152). All participants provided informed consent prior to participation.

## Conflicts of Interest

The authors declare no conflicts of interest.

## Supporting information


Supporting Information S1

Supporting Information S2

## Data Availability

The data that support the findings of this study are available from the corresponding author upon reasonable request.
