# Supplementary material for: Clinician Perspectives on Prescribing Restrictions and Opioid Pain Management in Australian Podiatric Practice: A Qualitative Study
Source: J Foot Ankle Res. 2026 Aug 19;19(3):e70202. doi: 10.1002/jfa2.70202 (PMC13490340; doi:10.1002/jfa2.70202)
Supplement: Supplementary file 1 [file JFA2-19-e70202-s001.docx]

## **Supplementary 2 – Profession-Specific Interview Guides**

**Semi-Structured Interview Guide: Pharmacists**

**Semi-Structured Interview Questions**

**Interviewer Instructions:** Read each question as written. Allow the participant to respond fully before proceeding. Provide definitions where necessary. Probe responses where appropriate. Maintain a neutral, non-leading tone throughout.

**Question 1:** What are your views on the current prescribing framework in which endorsed surgical podiatrists are authorised to prescribe paracetamol and codeine as a combination product and oxycodone but not tramadol or tapentadol, despite emerging evidence indicating that tapentadol may offer superior efficacy and safety for certain patient groups?

**Question 2:** What are your views on the current prescribing framework in which endorsed general podiatrists are authorised to prescribe paracetamol and codeine as a combination product, but not tramadol or tapentadol or oxycodone, given the well-documented variability in codeine metabolism due to genetic polymorphisms and its consequent unpredictable analgesic effect?

**Question 3:** In your professional opinion, how appropriate is oxycodone as a first-line opioid analgesic for surgical podiatrists managing acute, moderate nociceptive pain, particularly considering that current eTG guidelines favour tapentadol in many clinical contexts?

**Question 4:** In your professional opinion, how appropriate is paracetamol and codeine as a combination product as a primary analgesic option for podiatrists treating acute, moderate to severe nociceptive pain, particularly in light of its omission from current eTG recommendations?

**Question 5:** How do you feel about clinical situations in which evidence-based guidelines recommend one analgesic agent, but existing prescribing regulations compel clinicians to use potentially suboptimal or less evidence-supported alternatives?

**Question 6:** What are your thoughts on the use of fixed or static lists of scheduled medicines for specific healthcare professions, as opposed to a scope-of-practice-based prescribing model within an open or evolving formulary that accommodates emerging evidence?

**Question 7:** How do you view the role of pharmacists in advocating for equitable and clinically appropriate access to medicines, particularly in scenarios where existing prescribing policies may pose barriers to optimal patient care?

**Question 8:** In your view, how should evidence-based therapeutic guidelines, such as those published by the eTG, inform the development and updating of prescribing rights for various healthcare professions?

**Question 9:** What training, safeguards, and competencies do you believe would be necessary for an endorsed podiatrist, currently limited to prescribing paracetamol and codeine as a combination product, to be able to safely prescribe tramadol, tapentadol, and oxycodone for the evidence-based management of moderate to severe pain?

**Question 10:** What training, safeguards, and competencies do you believe would be necessary for an endorsed surgical podiatrist, currently limited to prescribing paracetamol and codeine as a combination product and oxycodone, to be able to safely prescribe tramadol and tapentadol for the evidence-based management of moderate to severe pain?

**Semi-Structured Interview Guide: Endorsed Surgical Podiatrists**

**Semi-Structured Interview Questions**

**Interviewer Instructions:** Read each question as written. Allow the participant to respond fully before proceeding. Probe responses where appropriate. Maintain a neutral, non-leading tone throughout.

**Question 1:** Can you tell me about your current practice setting and the typical patient population you serve?

**Question 2:** What are the most common types of pain you encounter in your patients?

**Question 3:** How do you currently manage moderate to severe pain in patients, particularly post-operatively or in acute pain conditions?

**Question 4:** Which analgesics do you frequently prescribe, and what are your views on their effectiveness?

**Question 5:** How do you feel about the restrictions on prescribing opioids in the list of scheduled medicines?

**Question 6:** Can you share any specific examples where the inability to prescribe tramadol affected a patient's pain management?

**Question 7:** What alternative strategies do you use when you believe tramadol would be the most appropriate medication but are unable to prescribe it?

**Question 8:** In your opinion, how does the inability to prescribe tramadol affect patient outcomes, particularly in terms of pain control and overall satisfaction with care?

**Question 9:** Have you ever had to refer a patient to another healthcare provider solely to obtain a tramadol prescription? If so, how did this impact the continuity of care?

**Question 10:** What additional training or education, if any, do you think surgical podiatrists would need to safely prescribe tramadol?

**Question 11:** If tramadol prescribing rights were expanded, how do you think it would change your practice?

**Question 12:** Is there anything else you would like to share about how the restriction on prescribing tramadol impacts your practice?

**Question 13:** Can you share any specific examples where the inability to prescribe tapentadol affected a patient's pain management?

**Question 14:** What alternative strategies do you use when you believe tapentadol would be the most appropriate medication but are unable to prescribe it?

**Question 15:** In your opinion, how does the inability to prescribe tapentadol affect patient outcomes, particularly in terms of pain control and overall satisfaction with care?

**Question 16:** Have you ever had to refer a patient to another healthcare provider solely to obtain a tapentadol prescription? If so, how did this impact the continuity of care?

**Question 17:** What additional training or education, if any, do you think surgical podiatrists would need to safely prescribe tapentadol?

**Question 18:** If tapentadol prescribing rights were expanded, how do you think it would change your practice?

**Question 19:** Is there anything else you would like to share about how the restriction on prescribing tapentadol impacts your practice?

**Question 20:** Do you feel that surgical podiatrists should have broader prescribing rights, including the ability to prescribe tramadol and tapentadol? Why or why not?

**Question 21:** What are your thoughts on the current legislative and regulatory environment governing podiatry practice in Australia, particularly regarding prescription authority?

**Question 22:** Are you involved in or aware of any advocacy efforts to expand surgical podiatrists prescribing rights?

**Question 23:** Do you have any suggestions or recommendations for policymakers regarding surgical podiatrists prescribing rights?

**Semi-Structured Interview Guide: Endorsed General Podiatrists**

**Semi-Structured Interview Questions**

**Interviewer Instructions:** Read each question as written. Allow the participant to respond fully before proceeding. Probe responses where appropriate. Maintain a neutral, non-leading tone throughout.

**Question 1:** Can you tell me about your current practice setting and the typical patient population you serve?

**Question 2:** What are the most common types of pain you encounter in your patients?

**Question 3:** How do you currently manage moderate to severe pain in patients, particularly post-operatively or in acute pain conditions?

**Question 4:** Which analgesics do you frequently prescribe, and what are your views on their effectiveness?

**Question 5:** How do you feel about the restrictions on prescribing tramadol in the list of scheduled medicines?

**Question 6:** Can you share any specific examples where the inability to prescribe tramadol affected a patient's pain management?

**Question 7:** What alternative strategies do you use when you believe tramadol would be the most appropriate medication but are unable to prescribe it?

**Question 8:** In your opinion, how does the inability to prescribe tramadol affect patient outcomes, particularly in terms of pain control and overall satisfaction with care?

**Question 9:** Have you ever had to refer a patient to another healthcare provider solely to obtain a tramadol prescription? If so, how did this impact the continuity of care?

**Question 10:** What additional training or education, if any, do you think podiatrists would need to safely prescribe tramadol?

**Question 11:** If tramadol prescribing rights were expanded, how do you think it would change your practice?

**Question 12:** Is there anything else you would like to share about how the restriction on prescribing tramadol impacts your practice?

**Question 13:** Can you share any specific examples where the inability to prescribe tapentadol affected a patient's pain management?

**Question 14:** What alternative strategies do you use when you believe tapentadol would be the most appropriate medication but are unable to prescribe it?

**Question 15:** In your opinion, how does the inability to prescribe tapentadol affect patient outcomes, particularly in terms of pain control and overall satisfaction with care?

**Question 16:** Have you ever had to refer a patient to another healthcare provider solely to obtain a tapentadol prescription? If so, how did this impact the continuity of care?

**Question 17:** What additional training or education, if any, do you think endorsed podiatrists would need to safely prescribe tapentadol?

**Question 18:** If tapentadol prescribing rights were expanded, how do you think it would change your practice?

**Question 19:** Is there anything else you would like to share about how the restriction on prescribing tapentadol impacts your practice?

**Question 20:** Do you feel that podiatrists should have broader prescribing rights, including the ability to prescribe tramadol and tapentadol? Why or why not?

**Question 21:** What are your thoughts on the current legislative and regulatory environment governing podiatry practice in Australia, particularly regarding prescription authority?

**Question 22:** Are you involved in or aware of any advocacy efforts to expand podiatrist prescribing rights?

**Question 23:** Do you have any suggestions or recommendations for policymakers regarding podiatrist prescribing rights and opioids?

**Semi-Structured Interview Guide: Dentists**

**Semi-Structured Interview Questions**

**Interviewer Instructions:** Read each question as written. Allow the participant to respond fully before proceeding. Probe responses where appropriate. Maintain a neutral, non-leading tone throughout.

**Question 1:** When prescribing for moderate to severe acute pain, you have authority to prescribe both oxycodone and tapentadol. Can you walk me through how you decide between these two options for a patient?

**Question 2:** What factors make you more likely to choose oxycodone over tapentadol, and vice versa?

**Question 3:** Describe a recent clinical situation where you considered both oxycodone and tapentadol. What ultimately guided your choice?

**Question 4:** How do you feel the competencies required to safely prescribe oxycodone differ to the competencies required to safely prescribe tapentadol?

**Question 5:** How do you think the training process for learning to prescribe oxycodone would differ from the training process for learning to prescribe both oxycodone and tapentadol?

**Question 6:** How do you feel learning to prescribe an individual "strong" opioid, such as oxycodone, would prepare a clinician to prescribe other opioids from the same class, such as tapentadol?

**Question 7:** From a patient safety perspective, if you had to recommend one of these medications to a colleague who was unfamiliar with both, what would guide your recommendation?

**Question 8:** How do you think we could educate a clinician who can legally prescribe oxycodone, to become a competent prescriber of tapentadol?

**Question 9:** For acute moderate pain management, how do you approach the choice between codeine and tramadol?

**Question 10:** What patient characteristics make you favour tramadol over codeine, and what makes you choose codeine over tramadol?

**Question 11:** How important do you believe it is for prescribing authority to align with evidence-based therapeutic guidelines like the Therapeutic Guidelines?

**Question 12:** When evaluating the appropriateness of a healthcare professional's prescribing scope, what role should national therapeutic guidelines play in determining which medications they can prescribe?

**Question 13:** How do you view the appropriateness of prescribing medications that lack guideline support versus being restricted from prescribing guideline-recommended alternatives?

**Question 14:** If the eTG specifically recommends certain medications for pain management, how do you feel this should influence prescribing authority decisions at the policymaker level?

**Question 15:** How do you feel the competencies required to safely prescribe codeine differ to the competencies required to safely prescribe tramadol?

**Question 16:** How do you think the training process for learning to prescribe codeine would differ from the training process for learning to prescribe both codeine and tramadol?

**Question 17:** How do you feel learning to prescribe an individual "weak" opioid, such as codeine, would prepare a clinician to prescribe other opioids from the same class, such as tramadol?

**Question 18:** How do you think we could educate a clinician who can legally prescribe codeine, to become a competent prescriber of tramadol?

**Question 19:** Do you have any other comments that you believe may help us?
