# Supplementary material for: Clinician Perspectives on Prescribing Restrictions and Opioid Pain Management in Australian Podiatric Practice: A Qualitative Study
Source: J Foot Ankle Res. 2026 Aug 19;19(3):e70202. doi: 10.1002/jfa2.70202 (PMC13490340; doi:10.1002/jfa2.70202)
Supplement: Supplementary file 2 [file JFA2-19-e70202-s002.docx]

# **Supplementary 1 – Definitions/Key Terms**

1. **Endorsed General Podiatrist (EGP)** – An endorsed general podiatrist is a registered podiatrist whose registration with the Podiatry Board of Australia (PBA) has been endorsed under Section 94 of the Health Practitioner Regulation National Law as qualified to administer, obtain, possess, prescribe, sell, supply, or use Schedule 2, 3, 4, and 8 medicines for the treatment of podiatric conditions, as specified in the National Podiatry Scheduled Medicines List (NPSML). Endorsement is obtained via completion of an approved postgraduate qualification in podiatric therapeutics, supervised clinical practice, and portfolio assessment aligned with the NPS MedicineWise Prescribing Competencies Framework. Endorsement indicates qualification to prescribe; it does not itself confer the authority to do so, actual prescribing authority is granted separately by individual state and territory drugs and poisons legislation.
2. **Endorsed Surgical Podiatrist (ESP)** – An endorsed surgical podiatrist (previously titled podiatric surgeon, with the title change taking effect from October 2026 – Surgical Podiatrist has been chosen as the standardised term for this manuscript as it aligns with forward focusing terminology) is an EGP, who additionally holds a surgical specialist registration with AHPRA. Surgical training is completed via either a five-year *Doctor of Podiatric Surgery* (University of Western Australia) or a competency-based fellowship through the Australasian College of Podiatric Surgeons, qualifying the practitioner to independently perform elective and reconstructive foot and ankle surgery.
3. **National Podiatry Scheduled Medicines List (NPSML) Prescribing** – NPSML is a fixed, statutory formulary approved by the Podiatry Board of Australia that specifies the Schedule 2, 3, 4, and 8 medicines that endorsed podiatrists and endorsed surgical podiatrists are qualified to administer, obtain, possess, prescribe, sell, supply, or use in the treatment of podiatric conditions. NPSML prescribing refers to the practice of prescribing within the confines of this list, which is legislatively embedded and cannot be updated without formal regulatory amendment. The list is stratified by practitioner category: EGPs may prescribe medicines from the general section of the NPSML (including codeine-paracetamol combination products), while ESPs have access to an expanded set of medicines, including a Schedule 8 opioid (oxycodone).
4. **Scope-of-Practice Prescribing** – Scope-of-practice prescribing is a prescribing model in which a clinician's authority to prescribe is determined dynamically by their demonstrated competence, clinical training, and professional role, rather than by a fixed, legislatively enumerated list of approved medicines. Under this model, as practiced by Australian nurse practitioners and dentists, practitioners may prescribe any medicine that falls within their area of clinical expertise and for which they hold the requisite knowledge and skills, subject to standard regulatory and safety requirements.
5. **Medicines Scheduling** – Medicines scheduling in Australia is a national classification system that controls how medicines and chemicals are made available to the public, based on their risk, potential for misuse, and the level of oversight required for safe use. Administered by the Therapeutic Goods Administration (TGA), schedules are published in the Standard for the Uniform Scheduling of Medicines and Poisons (SUSMP), commonly called the Poisons Standard, and are given legal force through individual state and territory legislation.

A) Schedule 2 (S2) — Pharmacy Medicine: available without prescription from a pharmacy

B) Schedule 3 (S3) — Pharmacist Only Medicine: requires pharmacist advice but no prescription

C) Schedule 4 (S4) — Prescription Only Medicine: requires a valid prescription (e.g., tramadol, codeine combination products post-2018 upscheduling)

D) Schedule 8 (S8) — Controlled Drug: subject to additional state/territory controls on prescribing, supply, and record-keeping due to high potential for misuse or dependence (e.g., oxycodone, morphine)

E) Schedule 9 (S9) — Prohibited Substance: may only be used for research under strict authorization
